# Huge mucinous cystadenoma of the pancreas mistaken for a pseudocyst

**DOI:** 10.11604/pamj.2013.15.6.2494

**Published:** 2013-05-03

**Authors:** Iyiade Olatunde Olaoye, Micheal Dapo Adesina

**Affiliations:** 1 University Of Ilorin Teaching Hospital, Nigeria

**Keywords:** Cystic tumor, pancreas, cystadenoma, pseudocyst

## Abstract

Cystic tumors of the pancreas are rare and can be confused with pseudocysts.We present a 50 year old woman with a huge mucinous cystadenoma of the pancreas initially diagnosed and managed with a cystojejunostomy and cyst wall biopsy. She required another laparotomy and tumor excision after histological diagnosis. Sensitivity of radiological imaging in differentiating between cystic pancreatic tumors and pseudocysts is limited. Cyst wall histology is diagnostic and biopsy of cyst wall should be done in cases with inconclusive preoperative diagnosis or questionable operative findings.

## Introduction

Cystic Neoplasms of the pancreas may be mistaken for pancreatic pseudocyst. Management of a pancreatic cystic tumor as pseudocyst may result in infection of the tumor or formation of gastric ulcers. Cross sectional radiological imaging may not be specific. Analysis of cystic fluid aspirate has improved diagnosis of pancreatic cystic lesions significantly. Biopsy and histology of cyst wall is diagnostic and should be considered in cases with inconclusive preoperative diagnosis and questionable intra operative findings. We present a patient with a huge mucinous cystadenoma of the pancreas initially mistaken for a pancreatic pseudocyst.

## Patient and observation

**Figure 1:**
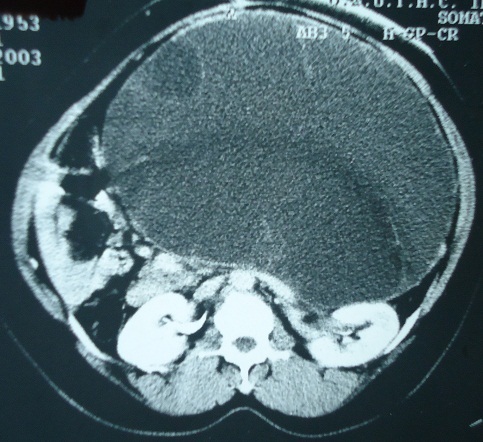
Huge mucinous cystadenoma of the pancreas CT Abdome

A 50 year old lady presented with four months history of back pain radiating towards the epigastrium. She also noticed a gradually increasing abdominal distension. She had weight loss. Bowel habits were normal. She had no previous history of abdominal pains or jaundice. She had an abdominal hysterectomy with left salphingo oophorectomy for uterine fibroids and left ovarian cyst two years before presentation. She had a huge firm non tender central abdominal mass occupying the upper 2/3 of the abdomen. Abdominal ultrasonography and CT scan (Figure 1) suggested a pancreatic pseudocyst. Fasting blood sugar and other relevant hematological parameters were normal. Laparotomy revealed a huge retroperitoneal cyst displacing the stomach upwards and the small bowel into the pelvis. The cyst had a thick wall and contained a thick viscid clear fluid. A biopsy of the wall of the cyst was taken and a cystojejunostomy fashioned. She had post-operative fever that was managed with parenteral antibiotics and was discharged eight (8) days after surgery.

Histology of cyst wall revealed Mucinous cystadenoma of the pancreas. She had another laparotomy and complete excision of an infected tumor. Tumor arose from the tail of pancreas and weighed about 3.6kg. Post-operatively, she had hypoproteineamia that improved before discharge. She was discharged fifteen days after the second surgery. She has been followed up for more than five years years and has remained well.

## Discussion

Cystic neoplasms of the pancreas are rare and comprise 10% to 15% of pancreatic cystic masses and only 1% of pancreatic cancers [1]. They are slow growing indolent tumors with low-grade malignant potentials and are mostly seen in middle aged women [2]. Commonly asymptomatic, they sometimes reach large sizes prior to diagnosis. Routine use of abdominal ultrasound, CT and MR has led to an increase in detection of pancreatic cystic lesions and reduced the average size at diagnosis [3, 4]. Pancreatic cystic neoplasms consist of Mucinous cystic neoplasms which commonly arise from the body and tail of the pancreas, Serous Cystic neoplasms that are almost always benign, Intraductal papillary mucinous tumors (IPMT) and unusual cystic neoplasms including cystic islet cell tumors. Mucinous cystic neoplasms include mucinous cystadenomas (65%), proliferative cystic mucinous neoplasms (30%) and mucinous cystadenocarcinomas [5].

Misdiagnosis and maltreatment of pancreatic neoplasms as pseudocysts is not uncommon [6]. Lack of a history of trauma, chronic pancreatitis or a recent history suggestive of acute pancreatitis should raise a possibility of a cystic neoplasm of the pancreas. Imaging with CT and MRI may not be specific though MRI is more accurate at differentiating a cystic neoplasm from a pseudocyst. Percutaneous or endoscopic ultrasonography with guided aspiration and analysis of cystic fluid for amylase activity, carcinoembryonic antigen level, viscosity, presence of mucin, cytology, tumor markers and DNA analysis improves diagnosis significantly [3, 7]. Carcino-embryonic antigen level estimation can be very sensitive in diagnosis of mucinous cystic tumors [8]. These investigations were not available for this patient. Intraoperatively, cystic neoplasms are thick walled and usually contain clear fluid, while pseudocysts fluid is usually grey, opalescent and contain blood or necrotic debris. Biopsy of cyst wall with frozen section and histology is diagnostic [9]. Maltreatment of cystic tumors as pseudocysts lead to infection as occurred in our patient and persistent painful gastric ulcers. Complete excision is the treatment of Mucinous cystic neoplasms of the pancreas because of the possibility of malignancy [1, 7]. Serous cystadenomas may be managed expectantly with close follow up. Recent improvement in morbidity and mortality of pancreatic surgery has encouraged complete excision in patients with serous cystadenoma. Complications of tumor resection include pancreatic fistula, portal vein thrombosis, abscesses, and hemorrhage.

## Conclusion

Cystic neoplasms are rare but should be considered in cystic lesions around the pancreas. An absence of history of trauma or history suggestive of inflammation of the pancreas may suggest a cystic tumor necessitating a detailed preoperative evaluation. A biopsy and histology of cyst wall is diagnostic and is indicated in patients with inconclusive preoperative assessment or with questionable intraoperative findings.

## References

[1] Horvath KD, Chabot JA (1999). An aggressive resectional approach to cystic neoplasms of the pancreas. Am J Surg.

[2] Delcore R, Thomas JH, Forster J, Hermreck AS (1992). Characteristics of cystic neoplasms of the pancreas and results of aggressive surgical treatment. Am J Surg.

[3] Gonzalvez Gasch AM, Mirete Ferrer C (2005). A case of a solid renal mass together with a cystic pancreatic lesion in a 50-year-old patient. JOP.

[4] Engelbrecht M, Bradshaw J, Smithuis R Pancreatic Cystic Lesions - Diagnosis and management. http://rad.desk.nl/en/4ec7bb77267de#p4ec7bb772d8ba.

[5] Sarr MG, Kendrick ML, Nagorney DM, Thompson GB (2001). Cystic neoplasms of the pancreas: benign to malignant epithelial neoplasms. Surg Clin North Am.

[6] Ooi LL, Ho GH, Chew SP, Low CH, Soo KC (1998). Cystic tumours of the pancreas: a diagnostic dilemma. Aust N Z J Surg.

[7] Al-Haddad M, El Hajj II, Eloubeidi MA (2010). Endoscopic ultrasound for the evaluation of cystic lesions of the pancreas. JOP.

[8] Cizginer S, Turner B, Bilge AR, Karaca C (2011). Cyst fluid carcinoembryonic antigen is an accurate diagnostic marker of pancreatic mucinous cysts. Pancreas.

[9] Warshaw AL, Rutledge PL (1987). Cystic tumors mistaken for pancreatic pseudocysts. Ann Surg.

